# FANS-UAS-assisted RIRS under local anesthesia: promising results, but are we ready for definitive conclusions?

**DOI:** 10.1097/JS9.0000000000005077

**Published:** 2026-03-18

**Authors:** Huiwen Luo, Lefeng Tian, Xirui Lu, Jing Yang

**Affiliations:** a NHC Key Laboratory of Nuclear Technology Medical Transformation, Mianyang Central Hospital, School of Medicine, University of Electronic Science and Technology of China, Mianyang, China; b State Key Laboratory of Environmental-friendly Energy Materials, Southwest University of Science and Technology, Mianyang Sichuan, China; c Operating Room, Mianyang Central Hospital, School of Medicine, University of Electronic Science and Technology of China, Mianyang, China; d Mianyang Key Laboratory of Anesthesia and Neuroregulation, Department of Anesthesiology, Mianyang Central Hospital, School of Medicine, University of Electronic Science and Technology of China, Mianyang, China

*Dear Editor,*

We read with great interest the recent article by Zhang *et al* entitled “FANS-UAS under local anesthesia for urinary stones with high risk–postoperative urosepsis,” published in the International Journal of Surgery. The authors should be commended for addressing a clinically challenging scenario – namely, the management of renal stones in patients with persistent asymptomatic bacteriuria or pyuria – and for exploring an innovative combination of flexible and navigable suction ureteral access sheath (FANS-UAS) with local anesthesia (LA) to potentially reduce postoperative infectious complications.

The study is notable for several strengths. First, the authors conducted a multicenter prospective trial in a relatively large cohort of high-risk patients (n = 395), which is commendable given the difficulty of enrolling such a population. Second, the reported postoperative fever rate of only 2.8% and the absence of urosepsis are encouraging, particularly in light of previous reports showing that postoperative infectious complications remain a major concern after RIRS, especially in patients with preoperative inflammatory urine findings^[^1–3^]^. Third, the concept of actively controlling intrarenal pressure using suction-assisted sheaths is supported by prior experimental and clinical evidence demonstrating its potential to reduce pyelovenous backflow and bacterial translocation^[^4,5^]^. In this regard, the authors’ work provides valuable clinical data extending this concept to a particularly high-risk population.

Nevertheless, several important issues merit further discussion.

First, the absence of a concurrent control group in the prospective cohort substantially limits causal inference. Although the authors reasonably argue that conventional UAS-based RIRS is not recommended in this setting, the current design makes it difficult to disentangle the relative contributions of (1) FANS-UAS, (2) local anesthesia, and (3) perioperative antibiotic and patient selection strategies to the observed low infection rate. Previous randomized and controlled studies have shown that different anesthesia modalities may influence postoperative immune function and complication profiles^[^6–8^]^, but whether LA per se, independent of surgical technique, reduces infectious complications after RIRS remains uncertain. A carefully designed comparative study – such as FANS-UAS under LA versus FANS-UAS under GA, or FANS-UAS versus conventional UAS under similar anesthetic conditions – would be necessary to support claims of superiority.

Second, the definition of “high-risk of postoperative urosepsis (HR-POU)” deserves further clarification. In the present study, HR-POU is defined as persistent asymptomatic bacteriuria or pyuria after antibiotic treatment. While multiple studies have shown that preoperative bacteriuria, pyuria, or inflammatory urine findings are associated with an increased risk of postoperative fever or infectious complications after RIRS^[^9,10^]^, current EAU and International Alliance of Urolithiasis guidelines still distinguish these conditions from overt uncontrolled infection, which remains a contraindication to surgery^[^1,11^]^. Therefore, the population studied here represents a selected high-risk but still clinically stable group, and the results should not be extrapolated to patients with active urosepsis or uncontrolled infection. This distinction is crucial to avoid potential misinterpretation and overly aggressive clinical adoption.

Third, the assessment of infectious outcomes relies mainly on postoperative fever and short-term laboratory markers. While fever is a pragmatic and commonly used endpoint, it is a relatively nonspecific surrogate. Prior registry data and systematic reviews have emphasized the importance of standardized reporting of infectious complications, including sepsis, SIRS, and culture-proven infections^[^3,12^]^. A more granular classification of infectious events and longer follow-up would further strengthen the conclusions regarding safety.

Finally, although the authors present intriguing immunological data suggesting better preservation of NK cells and cytotoxic T cells under LA, these findings are exploratory. The causal link between these immunological differences and the reduced infection rate remains speculative, as acknowledged by previous perioperative immunology studies^[^7,13^]^. Future mechanistic or randomized studies would be required to confirm this hypothesis.

In summary, Zhang *et al* are to be congratulated for proposing an innovative and potentially valuable strategy for managing a difficult subgroup of stone patients. Their work provides important preliminary evidence that FANS-UAS-assisted RIRS under LA is feasible and appears safe in selected high-risk patients with persistent urinary tract inflammation. However, given the single-arm design and the specific definition of HR-POU, the current results should be interpreted as hypothesis-generating rather than definitive. Further controlled and preferably randomized studies are warranted to clarify the independent contributions of suction technology and anesthesia modality, to better define the optimal target population, and to confirm whether this approach truly reduces clinically meaningful infectious complications.

## Data Availability

All data analyzed or generated during this study are included in the article.
